# Calcination Effects on 3D-Printed Phosphate-Activated Geopolymer Lattices: Structure, Strength, and Stability in Acidic Media

**DOI:** 10.1021/acsomega.6c04848

**Published:** 2026-08-04

**Authors:** Gabriel Tochetto, Arielle Cristina Fornari, Gean Delise Leal Pasquali, Dachamir Hotza, Maria Eliza Nagel-Hassemer, Paolo Colombo

**Affiliations:** † Department of Sanitary and Environmental Engineering, 28117 Universidade Federal de Santa Catarina, Florianópolis, Santa Catarina 88040-900, Brazil; ‡ Department of Industrial Engineering, Universita Degli Studi di Padova, Padova, Veneto 35122, Italy; § Department of Environmental and Sanitary Engineering, 232192 Universidade Federal da Fronteira Sul, Erechim, Rio Grande do Sul 99700-970, Brazil; ∥ Department of Chemical Engineering and Food Engineering, 28117 Universidade Federal de Santa Catarina, Florianópolis, Santa Catarina 88040-900, Brazil

## Abstract

Phosphate-activated geopolymers (PAGPs), a class of acid-activated rather than alkali-activated binders, are promising monolithic materials, yet optimizing porosity while maintaining stability in highly acidic waters remains challenging. Here, a metakaolin–phosphoric acid geopolymer ink containing laponite and Pluronic F127 (PLU) was formulated for direct ink writing (DIW) and printed as lattice monoliths (15 × 15 × 8 mm). A calcination window (350–550 °C) was applied to remove the PLU template and to elucidate how thermal severity governs phase/bond evolution, pore features, mechanical integrity, and performance in a multicomponent synthetic acid water (pH 2.96) containing Fe, Al, Zn, Mn, and Cu. XRD/FTIR indicated progressive template removal, dehydration, and phosphate-network consolidation with increasing temperature, consistent with structural evolution reported for acid-based geopolymers. Texturally, calcination strongly increased bulk porosity (45% to 61–63%) and promoted mesopore “unblocking” (largest BJH pore diameter and pore volume at 450 °C), whereas the specific surface area (SSA) increased only modestly (4.89 to 6.53–6.87 m^2^ g^–1^), highlighting that the dominant porosity changes occurred at larger length scales not fully captured by N_2_ physisorption. Mechanically, a pronounced strength–porosity trade-off was observed: 350 °C increased compressive strength (13.72 ± 0.33 → 27.16 ± 0.89 MPa), while ≥450 °C produced highly porous lattices but reduced strength to ∼5–6 MPa. In acidic conditions, calcination did not uniformly reduce leaching/enhance removal for all target metals; however, it consistently reduced the release of framework/trace species (notably Al, Ni, and V), evidencing improved chemical stability under acidic exposure. Overall, calcination can be tuned either toward higher robustness (350 °C) or higher porosity with reduced leaching (≥450 °C), depending on the intended operating constraints in acidic remediation.

## Graphical abstract

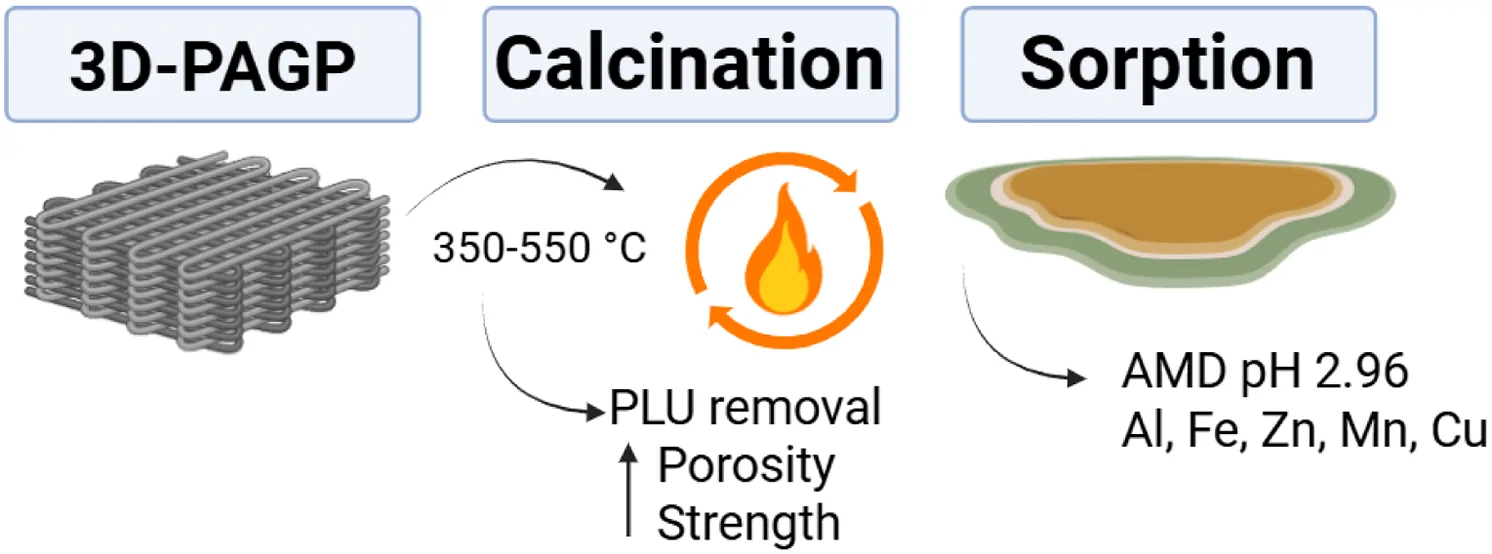

## 1 Introduction

Geopolymers are attractive candidates for water treatment because they are low-cost, intrinsically porous inorganic binders derived from aluminosilicate precursors and can be engineered into chemically durable monoliths.^1^ However, conventional geopolymer gels often exhibit limited intrinsic microporosity, which restricts their specific surface area (SSA) and, consequently, their applicability in water treatment.^2,3^ This limitation has driven the development of strategies to tailor geopolymer microstructures toward porous, high-SSA functional materials.^1,4^

One effective route is postsynthetic modification of alkali-activated geopolymers by combining acid etching with thermal treatment. Acid attack can rupture Si/Al bonds and promote the formation of surface silanol groups (Si–OH), evidencing network depolymerization,^5^ while heating removes adsorbed water and further alters pore architecture.^6^ Such combined treatments have been shown to increase SSA by several-fold (up to 190 m^2^ g^–1^) and to induce detectable mineralogical changes, including the emergence of new phases after modification, ultimately improving the suitability of geopolymers as for water treatment applications.^2^

In parallel, phosphate-based geopolymers (PAGPs) have gained attention due to their distinct Si–Al–P chemistry and promising thermal behavior relative to alkaline systems.^7^ Thermal analysis of phosphate-activated geopolymers commonly indicates endothermic events up to ∼300 °C associated with water removal and progressive structural reorganization,^8^ while XRD patterns often reflect predominantly amorphous Si–P/Al–P/Si–Al–P networks with possible crystallization of aluminophosphate phases (e.g., berlinite, AlPO_4_) depending on composition and thermal history.^9^

For porosity-oriented architectures, surfactant-assisted pore formation offers an additional lever to control open porosity and transport pathways.^10^ In phosphoric-acid systems, surfactant-based frothing using Triton X-100 has produced open-cell geopolymer foams with high total and open porosity (78% and 77%, respectively),^9^ underscoring the potential of surfactants to template interconnected macroporosity in phosphate-based matrices.

Additive manufacturing, and in particular direct ink writing (DIW), has recently been extended to geopolymers, enabling geometrically controlled, hierarchically porous monoliths that are difficult to obtain by casting.^11^ For phosphate-activated systems, extrudable inks with tailored rheology have been formulated and printed into self-supporting structures, and porous lattice architectures have been generated through filling strategies that vary the deposited filament width.^12,13^ This positions DIW-printed PAGPs as promising monolithic platforms for water treatment, where open, interconnected porosity and a stable framework are both required. More recently, a companion investigation on 3D-printed PAGP–PLU/AC composites further examined their structural stability and ion retention/release behavior under AMD-relevant acidic conditions, revealing selective Fe immobilization alongside framework dealumination as the two dominant, concurrent processes governing performance.^14^

Despite these advances, the coupled effect of thermal exposure and surfactant-modified microstructures remains insufficiently mapped for additively manufactured phosphate-based geopolymers, particularly when targeting porous lattice production and therefore the formation of micro/meso-pores. In geopolymers, temperature can simultaneously influence (i) consolidation and water removal, (ii) pore coarsening or collapse via densification, and (iii) chemical/mineralogical evolution detectable by FTIR and XRD, each of which directly impacts SSA and surface reactivity.

Therefore, this study explores the influence of thermal treatment on a 3D-printed PAGP modified with a surfactant, with the premise that calcination not only alters the material’s composition but also drives concomitant changes in texture, morphology and surface chemistry relevant to leaching/stability behavior. By systematically varying the temperature window from mild to aggressive conditions, we investigate how these processing variables impact surfactant removal, possible structural reorganization and the evolution of textural features, and how such changes ultimately relate to the metallic-ion leaching/release behavior. Correlating textural properties with chemical and mineralogical signatures, this work aims to elucidate the underlying structure–processing–performance relationships that govern the suitability of phosphate-based geopolymers for water treatment applications under acidic conditions. To the best of our knowledge, this is the first systematic assessment of how calcination severity controls template removal, multiscale porosity, mechanical integrity, and chemical stability of a surfactant-templated, additively manufactured PAGPs evaluated under simulated AMD. The specific advance is a processing, structure, and stability relationship that allows the calcination schedule to be selected according to whether mechanical robustness or higher porosity with reduced leaching is prioritized.

## 2 Materials and Methods

### 2.1 Preparation of the Geopolymer Printing Slurry

The slurries were prepared by a stepwise high-shear mixing protocol (2200 rpm). First, Pluronic F127 (PLU; 0.25 g; Sigma-Aldrich, DE), a nonionic poly­(ethylene oxide)-poly­(propylene oxide)-poly­(ethylene oxide) triblock copolymer was dissolved in 10 M of H_3_PO_4_ (20 g; Sigma-Aldrich, DE) for 5 min to ensure homogeneous dispersion of the surfactant prior to powder addition. PLU self-assembles into micelles that act as a sacrificial pore template and disperses readily in the acidic phosphate medium, and its thermal decomposition window (about 300 to 400 °C) is compatible with the calcination schedule used here. The PLU content of 0.7 wt % corresponds to the optimum identified in our previous study of this DIW system,^13^ in which surfactant contents of 0, 0.7, and 1.4 wt % were screened by rheology; 0.7 wt % provided the highest viscosity at rest, the highest storage modulus within the linear viscoelastic region, and the fastest thixotropic recovery, offering the best compromise between pore templating and the maintenance of extrudable, shape-stable filaments. Then, metakaolin (MK; 13 g; Argical 1200S, Imerys S.A., FR) was incorporated and mixed for 15 min while the container was placed in an ice bath to limit temperature rise during the exothermic acid–solid interaction and maintain a stable rheology. Finally, Laponite (LP; 1.5 g; BYK Additives, GR) was added and mixed for an additional 15 min to tailor viscosity and structural recovery for extrusion printing.^12^ The resulting formulation was denoted PAGP and presented the following molar ratios and liquid-to-solid ratio: H_3_PO_4_/Al_2_O_3_ = 2.52, SiO_2_/Al_2_O_3_ = 2.38, H_2_O/H_3_PO_4_ = 2.81, and L/S = 1.36.

### 2.2 Direct Ink Writing of Geopolymer Lattices

The prepared slurries were transferred into a syringe and installed on a DIW Delta-type 3D printer (Delta Wasp 2040 Turbo, Wasproject, Italy) fitted with a pressure vessel and a screw-based extrusion unit. Printing was carried out in ambient air at room temperature, using a conical nozzle with an 840 μm outlet (Nordson Italia S.p.a., Italy). The printhead speed was set to 10 mm s^–1^, while the extrusion rate was tuned as needed to ensure a continuous filament deposition. Lattice specimens were fabricated by depositing parallel geopolymer filaments with a 1 mm strand-to-strand spacing. Successive layers were rotated by 90° relative to the previous layer, producing a regular grid architecture. The structures were generated using the Variable Bead Width Porous Filling (VBWPF) strategy,^15,16^ with overall dimensions of 15 mm × 15 mm and a height of 8 mm. Following printing, the samples were cured at 25 °C for 24 h to allow completion of the geopolymerization process.

### 2.3 Calcination Strategy for PLU Template Removal

In the calcination study, three thermal schedules were designed to remove the PLU surfactant template while probing how calcination severity affects the integrity and porosity of the phosphate-activated geopolymer. The selected parameters (temperature, plateau time, and heating rate) are summarized in Table 1, which reports the thermal treatment conditions applied for each sample.

**Table 1:** Thermal Treatment Parameters Used for Surfactant Template Removal (T – Temperature; PT – Plateau Time; HR – Heating Rate)

Sample	T (°C)	PT (h)	HR (°C min^–1^)
PAGP–PLU_0.25/350 °C_	350	6	1
PAGP–PLU_0.25/450 °C_	450	4	2
PAGP–PLU_0.25/550 °C_	550	2	5

As shown in Table 1, all treatments were conducted at temperatures above 300 °C, since this range is typically required to achieve the full decomposition/combustion of organic species and, in particular, to ensure the effective removal of surfactant-derived residues from the inorganic matrix. The three calcination schedules were selected to represent increasing thermal severity, allowing the evaluation of the balance between template removal and preservation of the geopolymer porous structure and phosphate-based binding phases.^17–19^

i. 350 °C (6 h, 1 °C min^–1^) was selected as a mild calcination condition. The longer plateau time combined with a slow heating rate promotes gradual degradation of the organic template while minimizing thermal stress and preserving the printed microstructure.
ii. 450 °C (4 h, 2 °C min^–1^) represents an intermediate treatment condition intended to promote efficient surfactant removal while maintaining the structural integrity of the geopolymer matrix.
iii. 550 °C (2 h, 5 °C min^–1^) corresponds to a more severe calcination condition aimed at ensuring complete elimination of organic residues, while also enabling the evaluation of possible thermal effects on the geopolymer network, such as pore coarsening, matrix densification, or phase transformations.

Temperature was treated as the primary variable defining the severity of each schedule, while plateau time and heating rate were coadjusted following common debinding practice. We acknowledge that this design does not isolate the individual contribution of each parameter, and the schedules are therefore compared as integrated severity levels rather than in a one-factor-at-a-time manner.

Overall, by comparing the three schedules in Table 1, the study evaluates the trade-off between *(i) maximizing template removal* and *(ii) preserving the porous structure and chemistry* required for chemical stability and performance in acidic media.

### 2.4 Structural, Textural, and Mechanical Characterization

Compressive strength was measured using a universal testing machine (UTM, Quasar 25, Galdabini, IT) under uniaxial compression, with results expressed in megapascals (MPa). The reported values are the average of 5 tests using lattices with 15 × 15 × 8 mm. Specific surface area (SSA) and pore size distribution were determined using nitrogen adsorption via the Brunauer–Emmett–Teller (BET) and Barrett–Joyner–Halenda (BJH) methods in an automatic analyzer (Autosorb I, Quantachrome, US). Microstructural analysis included scanning electron microscopy (SEM, Cube II, EmCrafts, KR) coupled with energy-dispersive spectroscopy (EDS, AZtecOne, Oxford Instruments, UK) to observe surface morphology and elemental composition. Crystalline phases were identified using X-ray diffraction (XRD, MiniFlex600, Rigaku, JP), while Fourier transform infrared spectroscopy (FTIR, Spectrum Two, PerkinElmer, US) revealed functional groups. These combined tests provided a comprehensive understanding of the material’s properties for targeted applications.

### 2.5 Metal Ion Leaching Experiments under Simulated AMD

To assess the remediation performance and ion affinity of the 3D-printed geopolymer lattices under AMD-like conditions, a multicomponent metal solution was prepared using Fe_2_(SO_4_)_3_·3H_2_O (Fe, Dinâmica, BR), Al_2_(SO_4_)_3_·16H_2_O, (Al, Dinâmica, BR), ZnSO_4_·7H_2_O (Zn, Vetec, BR), MnSO_4_·2H_2_O (Mn, Vetec, BR), CuSO_4_·5H_2_O (Cu, Vetec, BR). The salts were dissolved in distilled water, and the pH of the resulting solution was adjusted to 2.96 ± 0.06 using HCl (0.1 M, Neon, BR) to maintain metal solubility. The pH was adjusted to 2.96 to represent a realistic acidic AMD condition consistent with the broader remediation project and to keep the target metals in solution. Because the salts used were the sulfates of Fe, Al, Zn, Mn, and Cu, the anionic background is sulfate-dominated; the total sulfate concentration was not quantified in this work. The measured initial concentrations were 6.19 mg L^–1^ Fe, 11.99 mg L^–1^ Zn, 3.23 mg L^–1^ Cu, 3.16 mg L^–1^ Mn, and 0.67 mg L^–1^ Al (Table 3), selected to emulate a moderately loaded multicomponent AMD.^3,13^ Each PAGP–PLU lattice was then contacted with 50 mL of the multicomponent solution in an Erlenmeyer flask (one lattice per reactor). The flasks were continuously agitated at 150 ± 5 rpm and 25 ± 2 °C for 15 h. Each condition was evaluated in duplicate. After the contact period, the suspensions were filtered to terminate the solid-solution interaction, and the final pH was recorded. The pH was not actively buffered; it was monitored before and after contact and remained within 2.8 to 2.9 throughout the experiments. Metal concentrations in the filtrates (C15h) and also the original multicomponent solution (Ci) were quantified by inductively coupled plasma optical emission spectrometry (ICP–OES; ICPE-9820, Shimadzu, Japan), using argon (Air Liquide) as the carrier gas. Prior to analysis, all samples were additionally filtered through 0.45 μm polytetrafluoroethylene (PTFE, Filtrilo, BR) syringe filters. The equipment was calibrated using solutions with concentrations ranging from 10 to 200 μg L^–1^, prepared from the multielemental standard solution (Specsol, 10.00 ± 0.05 mg L^–1^).

## 3 Results and Discussion

### 3.1 Effect of Calcination on PAGP–PLU Composition

The compositional evolution evidenced by XRD and FTIR (Figure 1) directly supports the calcination rationale/hypotheses defined in Section 2.3 (Table 1). Because PLU typically undergoes its main thermal degradation in the 300–400 °C range,^20^ the selected schedules (350–550 °C) were expected to progressively eliminate the organic template and reduce carbonaceous residues,^19^ while simultaneously driving dehydration/condensation of the inorganic geopolymer network. Although thermogravimetric analysis was not available in our facilities, this decomposition window of PLU is well established in the literature,^19,20^ and the disappearance of the C–H stretching bands in FTIR (Figure 1B) provides direct evidence of template removal within the calcination range applied here.

**Figure 1:**
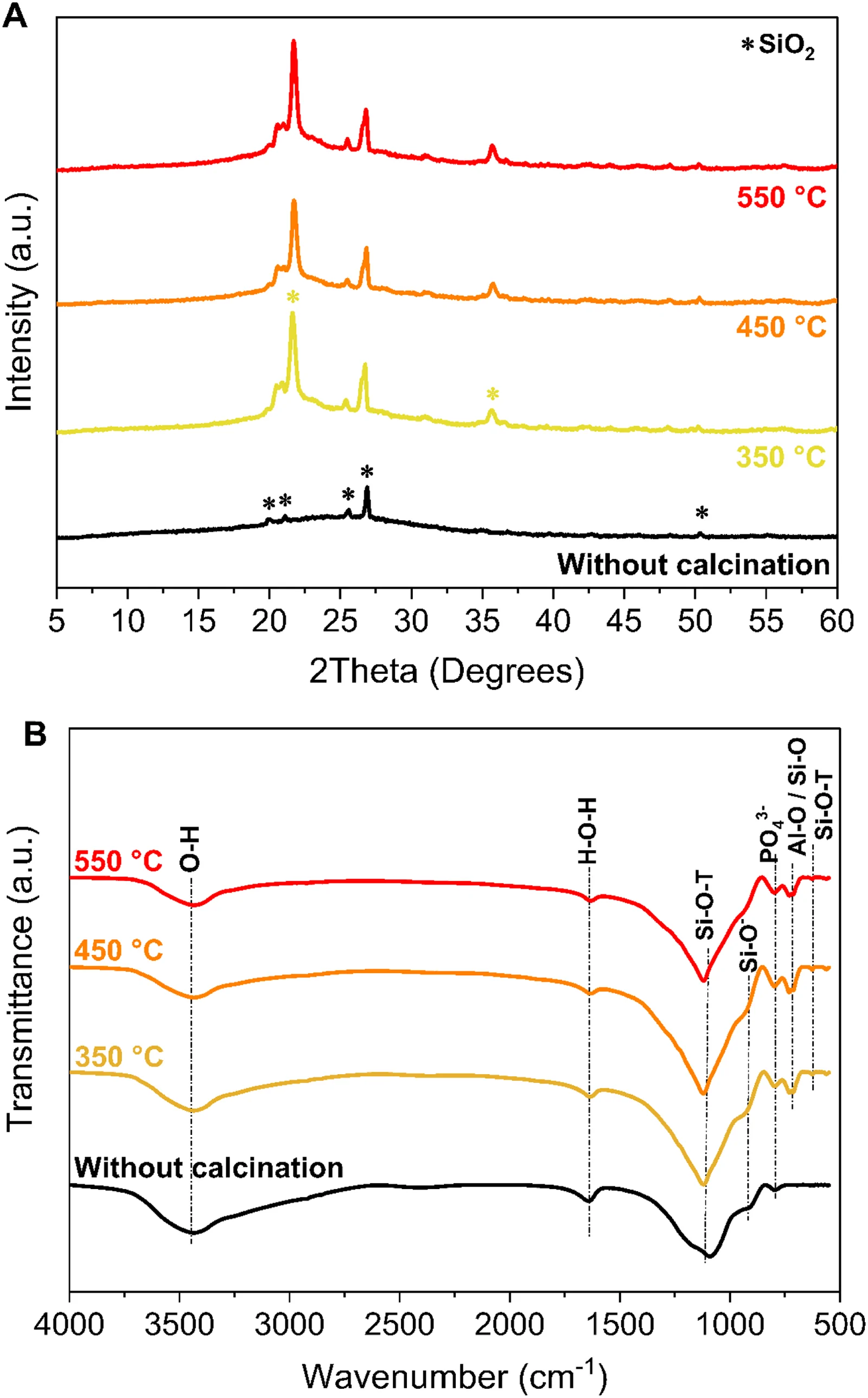
(A) XRD patterns and (B) FTIR spectra of PAGP–PLU_0.25_ before and after calcination at 350, 450, and 550 °C.

In XRD (Figure 1A), PAGP–PLU_0.25_ is dominated by a broad diffuse halo, consistent with a predominantly amorphous phosphate-activated geopolymer binder, with superimposed reflections attributable to crystalline impurities inherited from the metakaolin (typically quartz, titania, and minor phases).^7^ This behavior aligns with phosphate-based geopolymers, which commonly remain largely X-ray amorphous while retaining impurities-derived peaks; in contrast, pronounced crystallization (e.g., aluminum-phosphate/berlinite and related phases) is generally associated with more severe firing conditions (i.e., 200 °C).^12,21,22^ Upon calcination (350–550 °C), the inorganic reflections become more discernible relative to the diffuse background, which is consistent with organic-template burnout and progressive dehydration/condensation of the gel, both of which reduce diffuse scattering and enhance diffraction contrast.^23,24^ In situ XRD studies on surfactant-templated porous solids report a clear increase in scattering contrast upon template removal, supporting this interpretation.^24^ Importantly, within this moderate temperature window, the data are more indicative of structural relaxation/reorganization and densification than of a complete transformation into a highly crystalline ceramic phase,^9^ reinforcing that increased severity can alter the framework but does not necessarily fully recrystallize the binder under the selected schedules.

FTIR (Figure 1B) shows progressive dehydration/consolidation upon calcination. The broad O–H stretching envelope (3165–3660 cm^–1^) and the H–O–H bending band (1630–1640 cm^–1^, with possible contribution from P–O–H)^25,26^ are strongest in the noncalcined sample and attenuate stepwise at 350 °C, 450 °C and most markedly at 550 °C, indicating gradual removal of molecular/physically bound water during thermal consolidation.^26–28^ The effective removal of the PLU template is evidenced by the disappearance of the characteristic C–H stretching shoulders observed in the 2850–2980 cm^–1^ range of the noncalcined spectrum, alongside a discernible narrowing of the main Si–O–T/P–O–T envelope at 1100 cm^–1^ as the overlapping C–O–C ether contributions from the polymer are eliminated.^29^ In the fingerprint region (900–1100 cm^–1^), the spectra are dominated by overlapping Si–O–T (T = Si, Al) and phosphate-related (P–O/P=O and P–O–T) vibrations typical of metakaolin–phosphoric acid binders.^12^ With calcination, this complex band becomes more defined and its relative components (including the 930 cm^–1^ contribution) become more discernible,^26^ consistent with temperature-driven rearrangement/condensation of mixed aluminosilicate–phosphate environments.^28^ Finally, bands in the 800–500 cm^–1^ region (assigned to Al–O/Si–O and phosphate/network modes)^30^ are more clearly resolved in the calcined samples, particularly at 550 °C.

Figure 1 provides mechanistic confirmation of the experimental design in Table 1: *(i) at 350 °C*, the spectra/patterns are consistent with substantial but potentially incomplete template removal alongside dehydration/condensation; *(ii) at 450 °C*, further reduction of hydroxyl/water contributions and clearer inorganic features are consistent with more complete PLU elimination while maintaining a largely geopolymeric matrix; and *(iii) at 550 °C*, continued condensation and subtle structural reorganization are suggested, matching the hypothesis that aggressive calcination can intensify inorganic rearrangement in phosphate-bearing binders. Overall, calcination therefore modifies the composition of PAGP–PLU in a chemically meaningful way, by removing the PLU organic fraction and driving phosphate-network consolidation, while retaining a predominantly amorphous geopolymer character up to 550 °C.

### 3.2 Effect of Calcination on Texture, Morphology, and Strength

The effect of calcination on the dimensional stability, bulk porosity, pore accessibility, and mechanical performance of PAGP–PLU_0.25_ is summarized in Table 2 and supported by optical images and micrographics observations. Overall, increasing the calcination severity primarily promoted the removal of the PLU-derived organic fraction and other volatiles, leading to pronounced changes in apparent density/porosity and in the mesopore domain (BJH), while the intrinsic density of the inorganic phase increased only slightly. This trend is consistent with the general understanding that thermal template removal is a stepwise process, in which organics are progressively decomposed/oxidized and pores become gradually free and accessible.^18,31^ Optical micrographs of the printed lattices are shown in Figure 2, confirming the regular grid architecture and the dimensional fidelity of the deposited filaments.

**Figure 2:**
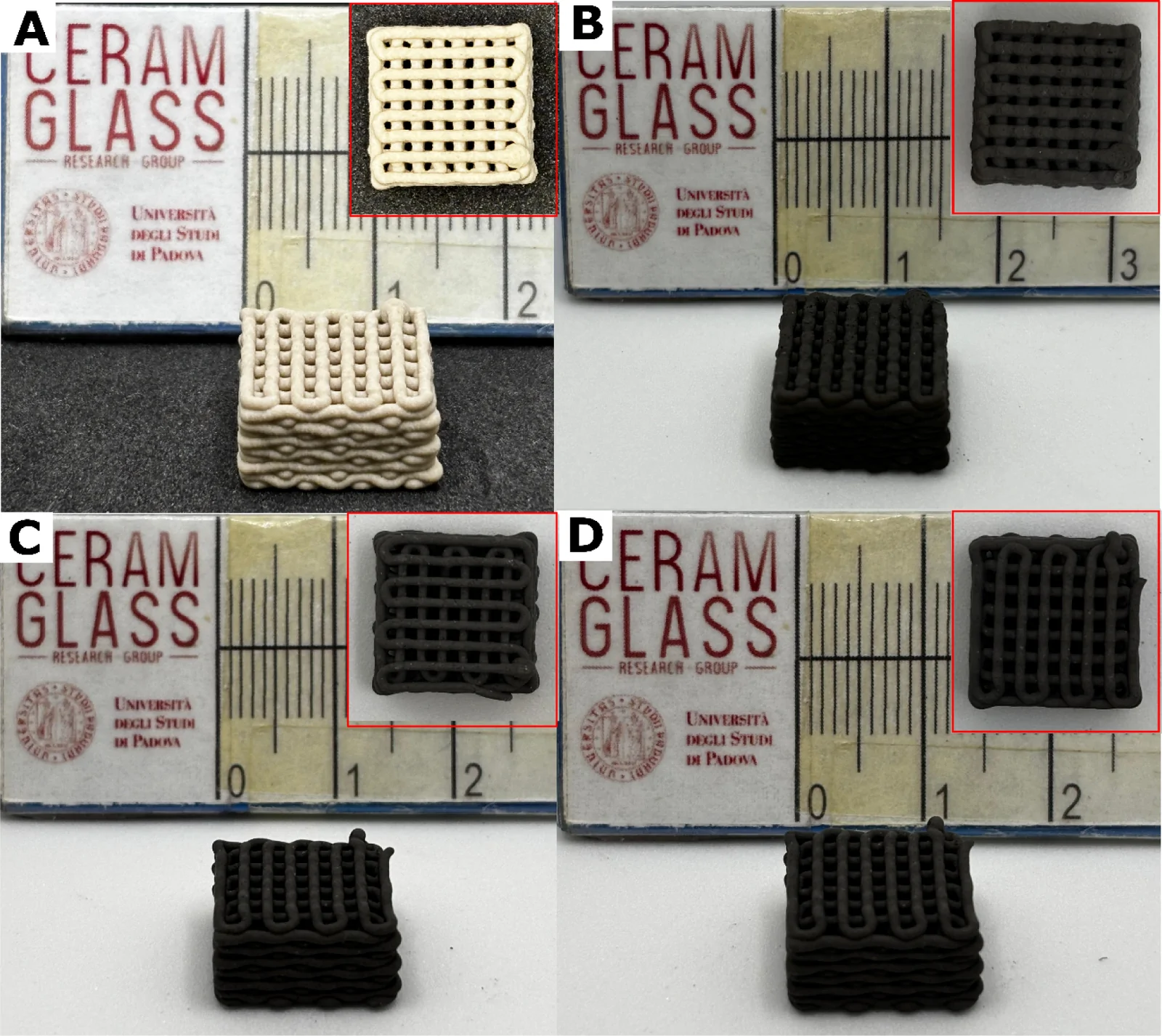
Optical micrographs of the printed (A) PAGP–PLU_0.25_, (B) PAGP–PLU_0.25/350 °C_, (C) PAGP–PLU_0.25/450 °C_, (D) PAGP–PLU_0.25/550 °C_ lattices showing the grid architecture.

**Table 2:** Effect of Calcination Temperature (350–550 °C) on Shrinkage, Density/Porosity, and Textural Properties of PAGP–PLU_0.25_ (CS – Compressive Strength; SSA – Specific Surface Area; PD – Pore Diameter; PV – Pore Volume)

Parameter	PAGP–PLU_0.25_	PAGP–PLU_0.25/350 °C_	PAGP–PLU_0.25/450 °C_	PAGP–PLU_0.25/550 °C_
Shrinkage (Model 15 × 15 × 8)				
L_1_ (mm)	13.78 ± 0.68	13.97 ± 0.12	13.67 ± 0.39	13.61 ± 0.29
Variation (%)	8.13	6.87	8.87	9.30
L_2_ (mm)	13.91 ± 0.43	14.06 ± 0.04	13.54 ± 0.27	13.55 ± 0.26
Variation (%)	7.25	6.27	9.73	9.67
h (mm)	7.61 ± 0.80	7.68 ± 0.27	7.96 ± 0.05	7.51 ± 0.09
Variation (%)	4.88	4.00	0.50	6.19
Physical properties				
ρ_true_ (g cm^–3^)	2.217 ± 0.001	2.348 ± 0.004	2.374 ± 0.003	2.381 ± 0.004
ρ_apparent_ (g cm^–3^)	1.220 ± 0.075	1.242 ± 0.072	0.883 ± 0.055	0.931 ± 0.038
Weight (g)	1.78 ± 0.06	1.87 ± 0.11	1.30 ± 0.08	1.29 ± 0.12
Porosity (vol%)	45.0 ± 3.38	47.1 ± 3.1	62.8 ± 2.3	60.9 ± 1.6
CS (MPa)	13.7 ± 0.3	27.2 ± 0.9	5.6 ± 0.3	6.0 ± 0.6
SSA (m^2^ g^–1^)	4.89	6.87	6.84	6.53
PD (nm)	10.74	12.8	17.1	14.2
PV (cm^3^ g^–1^)	1.31 × 10^–2^	2.21 × 10^–2^	2.93 × 10^–2^	2.33 × 10^–2^

From a geometrical standpoint, the printed lattices retained their architecture after calcination, exhibiting linear dimensional variations of the same order (6–10% in L_1_/L_2_) across all processing conditions (Table 2). Notably, the 450 °C schedule provided the best height preservation (h variation 0.5%), suggesting a more favorable balance between organic removal and thermal stress development within the struts. In contrast, the most aggressive treatment (550 °C) increased height variation (6.2%), consistent with intensified thermal shrinkage and microstructural rearrangements. These dimensional changes align with the accompanying evolution of bulk properties: while the true density increased from 2.217 to 2.38 g cm^–3^ (indicative of dehydration/condensation of the inorganic network), the apparent density decreased sharply at ≥450 °C (to 0.88–0.93 g cm^–3^), together with a marked mass loss (from 1.87 to 1.30 g). Because masses were measured on independently printed specimens, the slightly higher mean mass at 350 °C lies within the combined uncertainty of the uncalcined value and does not indicate a true mass gain; the pronounced decrease at and above 450 °C is well beyond this scatter and reflects template and volatile removal. As a consequence, total porosity rose from ∼45 vol% (uncalcined) to ∼63 vol% at 450 °C and remained high (∼61 vol%) at 550 °C (Table 2), highlighting extensive template burnout and pore opening. Such mass-loss-driven porosity development is coherent with the thermal decomposition behavior of PLU-based binders/templates, whose main degradation typically occurs in the ∼300–400 °C range, thereby creating additional void space upon removal.^19^

However, the BET/BJH data indicate that calcination primarily improved mesopore accessibility rather than generating a substantial increase in surface area (as observed in Figure 3). The uncalcined PAGP–PLU_0.25_ already presented measurable mesoporosity, which is consistent with the porous strut cross sections seen by SEM (Figure 4) and reflects processing-induced voids and template-related features. The corresponding N_2_ adsorption–desorption isotherms for all materials are presented in Figure 5. After calcination, the BET surface area increased only modestly (4.89 to 6.53–6.87 m^2^ g^–1^), indicating that the micro/mesoporous surface probed by N_2_ physisorption is comparatively resilient to thermal treatment and that the major porosity changes are dominated by larger voids (macroporosity/structural pores) not contributing significantly to the amount of surface area quantified by gas sorption measurements. The BJH pore diameter and pore volume exhibited a clearer dependence on temperature: 450 °C produced the largest mesopores (PD = 17.1 nm) and the highest BJH pore volume (PV = 2.93 × 10^–2^ cm^3^ g^–1^), suggesting a more effective template removal and pore unblocking at this intermediate condition (Table 1). At 550 °C, PD and PV decreased (14.2 nm; 2.33 × 10^–2^ cm^3^ g^–1^), pointing to partial pore-wall consolidation and/or textural relaxation with loss of the finer mesoporous contribution, a trend consistent with reports that more aggressive calcination can induce shrinkage/sintering effects and partial closure or coarsening of porous structures.^31^

**Figure 3:**
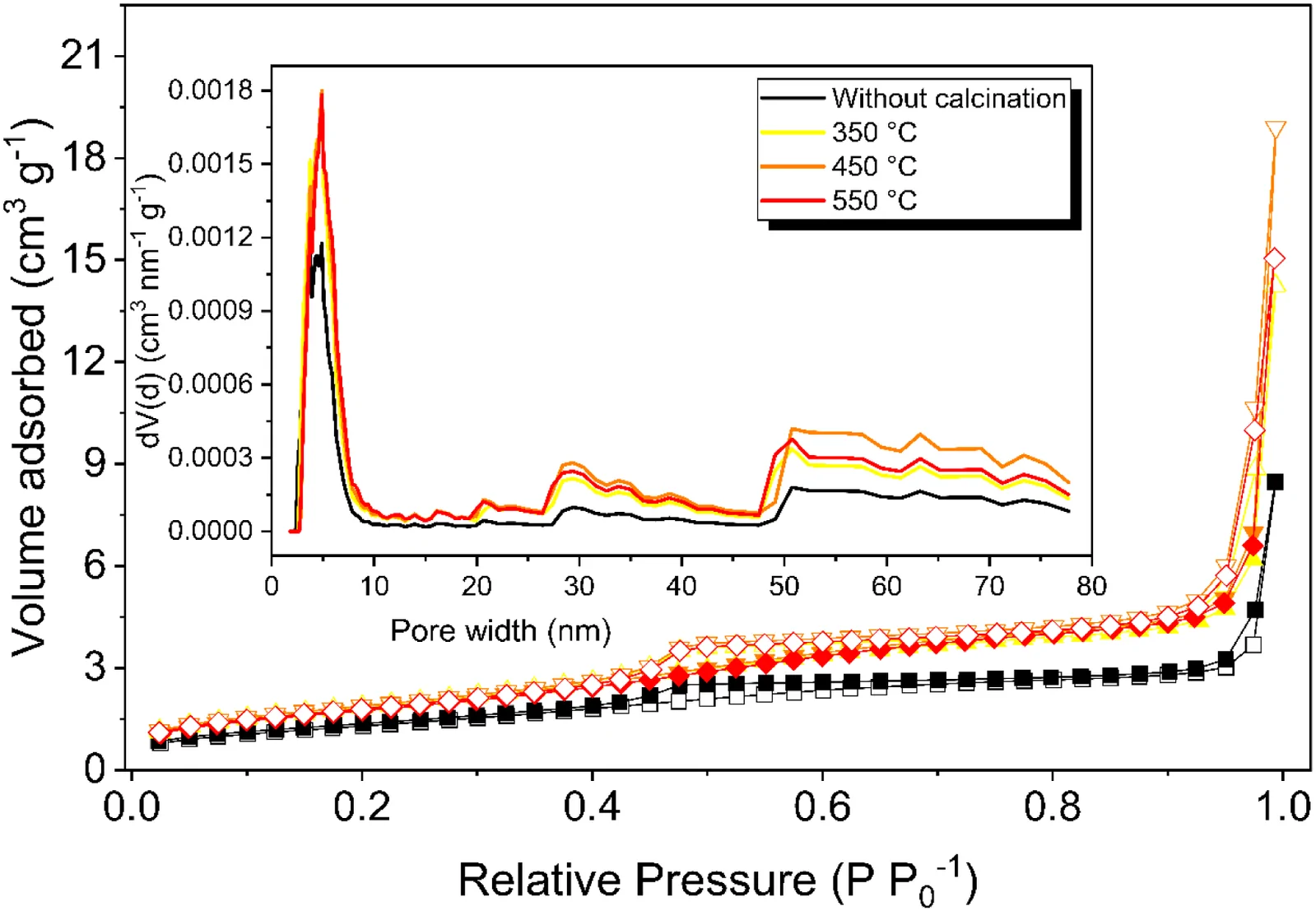
N_2_ adsorption–desorption isotherms of PAGP–PLU_0.25_ before and after calcination at 350, 450, and 550 °C.

**Figure 4:**
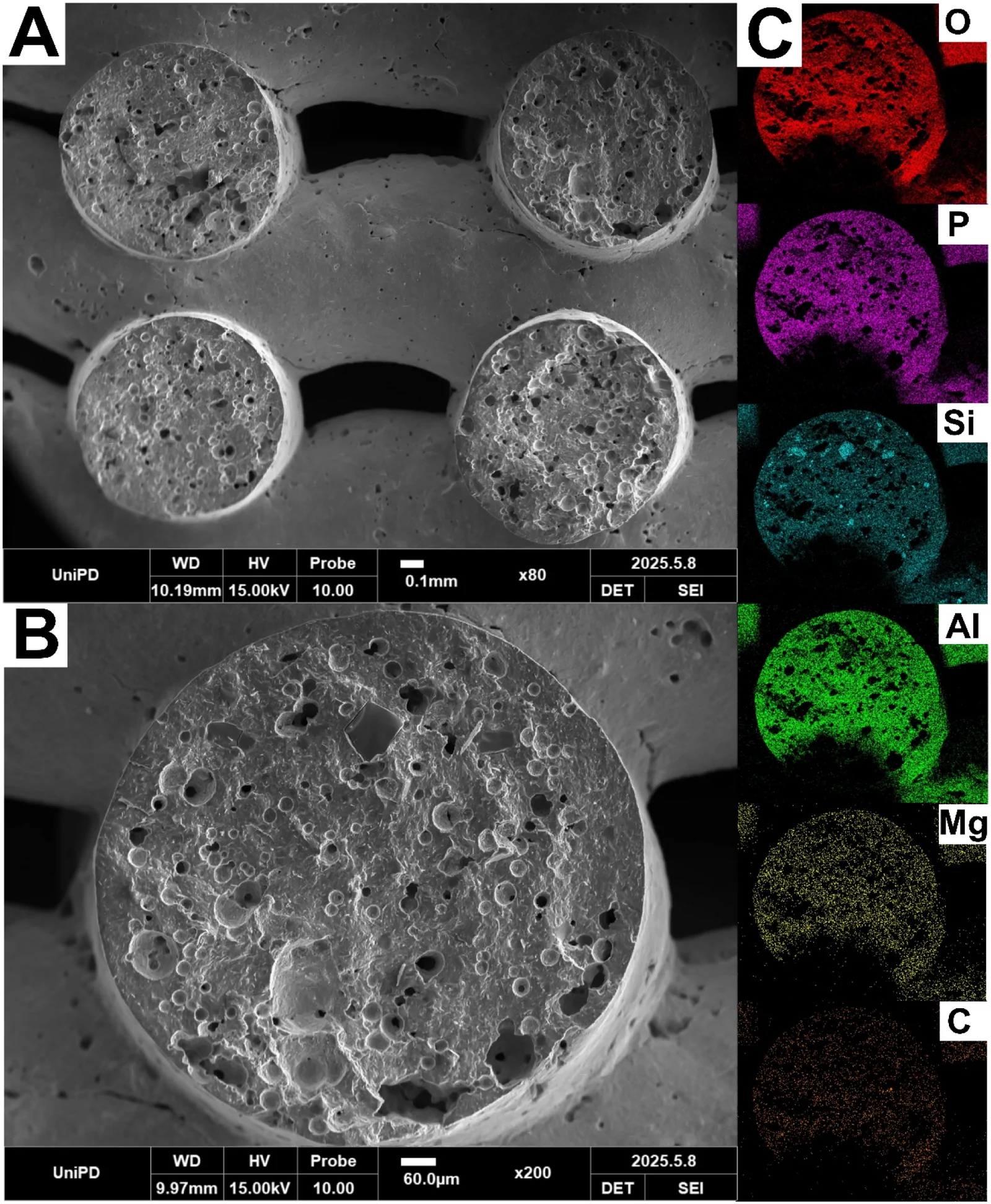
SEM micrographs of PAGP–PLU_0.25_ at (A) 80× and (B) 200× magnification, and (C) corresponding EDS elemental maps of the same region.

**Figure 5:**
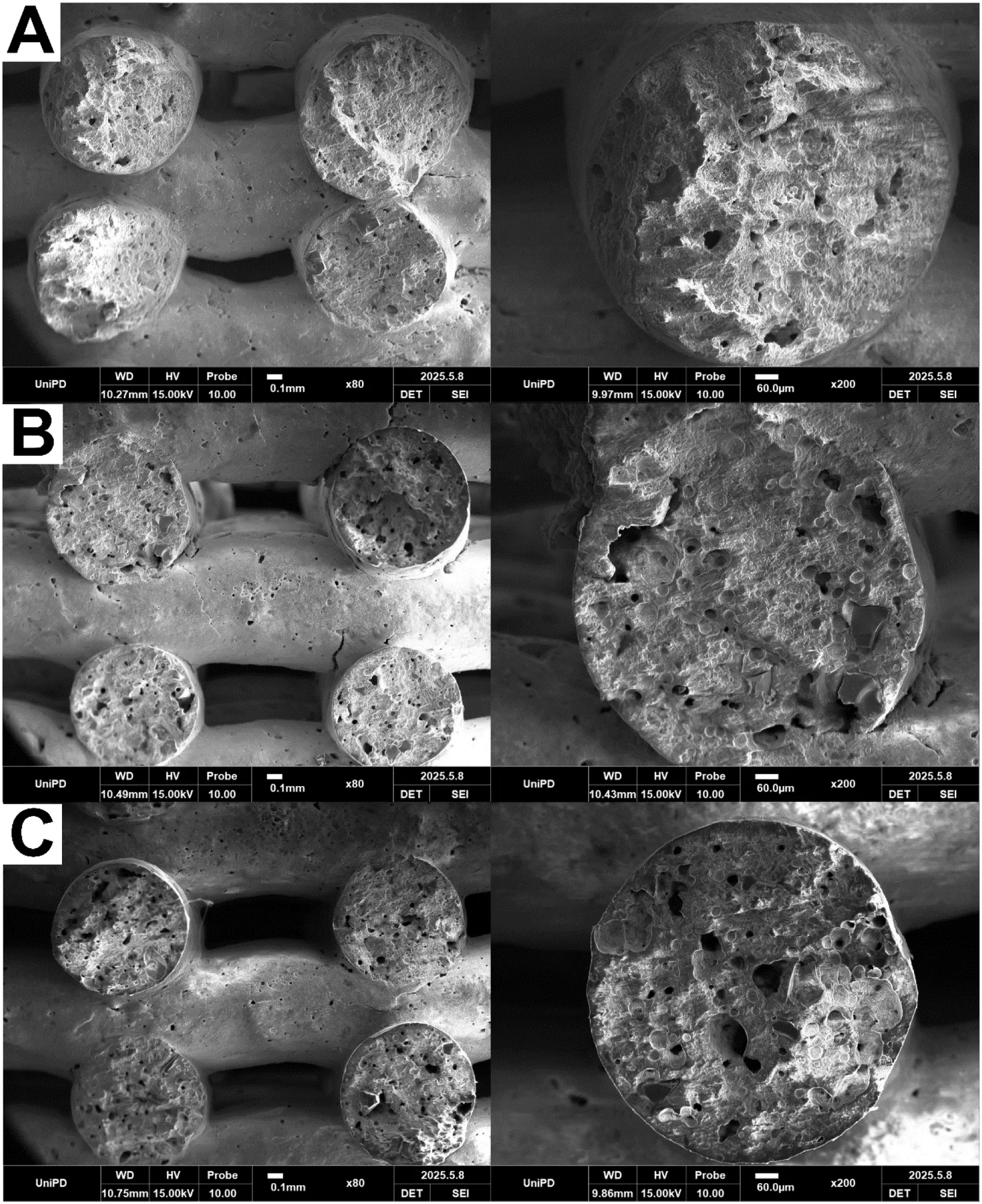
SEM micrographs of PAGP calcined at (A) 350 °C, (B) 450 °C, and (C) 550 °C at 80× and 200× magnification.

CS increased markedly after the mild treatment (from 13.72 to 27.16 MPa at 350 °C), consistent with partial template removal and dehydration/condensation effects that can stiffen the strut skeleton without yet creating excessive open porosity. However, at 450–550 °C the strength dropped sharply to ∼5–6 MPa (Table 2), despite the higher total porosity and larger BJH pore volume. This behavior is coherent with established structure–property relationships in porous solids, where strength scales strongly with relative density/porosity and is additionally penalized by larger defects, pore coalescence, and microcracking. Once porosity rises into the 60 vol% range and pores/voids become more connected, load-bearing cross-section and flaw tolerance decrease significantly.^32^

Importantly, the apparent discrepancy between the strong increase in bulk porosity (up to 63 vol%) and the modest changes in SSA can be rationalized by the multiscale pore system of DIW lattices: SEM-visible voids and interfilament spaces fall largely within the macropore regime, which is not fully captured by N_2_ physisorption that primarily characterizes micro/mesopores. This interpretation is reinforced by SEM: the uncalcined filaments show abundant rounded voids and a porous cross-section (Figure 4A–B), while calcined samples maintain a porous morphology with more open features at higher temperature (Figure 5A–C). EDS mapping of the uncalcined sample indicates a broadly homogeneous distribution of O, P, Si, Al, and Mg (Figure 4C), supporting compositional uniformity within the struts; the detected carbon signal is consistent with residual organics prior to calcination and supports the role of thermal treatment in removing the template fraction. Finally, because phosphate-activated matrices can undergo temperature-driven phase rearrangements (e.g., evolution toward AlPO_4_/berlinite-related motifs upon heating), the slight reduction in BJH pore volume and SSA at 550 °C is also consistent with the onset of inorganic reorganization/densification in this temperature window, which may compete with porosity development.^22,33^

Overall, raising the calcination temperature clearly modified the material’s texture and morphology, mainly by increasing bulk porosity and reshaping the BJH mesopore domain. These changes resulted in a pronounced strength–porosity trade-off: the mild treatment at 350 °C improved compressive strength, whereas ≥ 450 °C maximized porosity but caused a sharp decrease in mechanical resistance.

### 3.3 Effect of Calcination on Metal Removal and Matrix Stability

The multicomponent simulated AMD (pH 2.96) represents a highly aggressive chemical environment for metal retention because *(i) proton competition and surface protonation* generally suppress cation retention at pH < 3, and *(ii) the high ionic strength/complexation* (e.g., sulfate complexes) increases competition for binding sites. Under such conditions, apparent metal removal behavior can be strongly coupled to matrix stability (i.e., dissolution/leaching of the solid) rather than only interfacial uptake. This duality is well recognized in AMD remediation systems and in geopolymer-based materials, where low pH can both hinder adsorption and promote binder dissolution.^34,35^

Table 3 shows several elements for which the metal concentration after contact with the lattice (C15h) is much higher than the one before contact (Ci), C15h ≫ Ci, notably Al, Ba, Ni, V, Cr and, to a lesser extent, Zn and Mn. Because the starting solution was prepared from Fe/Al/Zn/Mn/Cu sulfates, the appearance (or large increase) of Ba, Ni, V, Cr is most plausibly attributed to leaching of trace constituents from the geopolymer matrix and/or from raw precursor impurities under acidic exposure, rather than to negative removal in the strict sense. This interpretation is consistent with pH-dependent leaching studies on metakaolin-based geopolymers showing enhanced release of major and trace elements under acidic conditions, including the potential mobilization of oxyanionic trace elements such as V.^35,36^

**Table 3:** Metal Ion Concentrations before (Ci) and after (C15h) Contact with PAGP–PLU Lattices Subjected to Different Calcination Strategies^a^

Metal ion	Ci	PAGP–PLU_0.25_ (C15h)	PAGP–PLU_0.25/350 °C_ (C15h)	PAGP–PLU_0.25/450 °C_ (C15h)	PAGP–PLU_0.25/550 °C_ (C15h)
Al (mg L^–1^)	0.67 ± 0.03	39.30 ± 0.42	23.40 ± 1.98	20.60 ± 0.28	10.55 ± 0.92
Ba (μg L^–1^)	0.38 ± 0.11	63.75 ± 0.71	48.52 ± 8.52	48.85 ± 1.13	41.85 ± 3.39
Cr (μg L^–1^)	7.20 ± 0.23	14.68 ± 0.25	10.00 ± 0.28	10.30 ± 0.57	8.95 ± 0.07
Cu (mg L^–1^)	3.23 ± 0.05	3.30 ± 0.05	3.13 ± 0.02	3.21 ± 0.04	3.28 ± 0.03
Fe (mg L^–1^)	6.19 ± 0.12	1.00 ± 0.05	1.93 ± 0.13	1.81 ± 0.13	1.95 ± 0.04
Mn (mg L^–1^)	3.16 ± 0.04	3.49 ± 0.08	3.41 ± 0.11	3.33 ± 0.00	3.36 ± 0.01
Ni (μg L^–1^)	7.97 ± 0.72	33.26 ± 5.90	32.68 ± 3.54	20.58 ± 0.84	18.13 ± 1.33
V (μg L^–1^)	0.33 ± 0.15	22.31 ± 0.74	10.70 ± 1.10	13.43 ± 0.49	7.87 ± 0.27
Zn (mg L^–1^)	11.99 ± 0.28	13.94 ± 0.49	13.69 ± 0.57	13.89 ± 0.57	13.44 ± 0.21

a Experimental conditions: one 3D lattice (single piece), 50 mL solution, 150 ± 5 rpm, 25 ± 2 °C, and 15 h contact time.

A key result is that calcination systematically reduced the magnitude of leaching for several species, particularly Al (from 39.30 to 10.55 mg L^–1^ from uncalcined to 550 °C), and also Ba (from 63.75 to 41.85 μg L^–1^), Ni (from 33.26 to 18.13 μg L^–1^) and V (from 22.31 to 7.87 μg L^–1^). This trend suggests that higher-temperature treatment improves the chemical stability of the phosphate-activated framework, likely through additional dehydration/condensation and the development of more water/acid-resistant Al–O–P linkages and/or AlPO_4_–like domains. Such temperature-driven evolution in aluminosilicate phosphate binders has been discussed in the context of SAP/ASP geopolymer chemistry and thermal stability.^37,38^

That said, the persistence of elevated Al leaching even after calcination (10.55 mg L^–1^ at 550 °C) indicates that acid dissolution of the Al present in the geopolymer matrix is not fully suppressed at pH ≈ 3, and some degree of framework or secondary-phase solubilization still occurs. This is consistent with the H^+^-driven dealumination mechanism described for phosphoric acid-activated geopolymers exposed to comparable acidic conditions, where protonation cleaves Si–O–Al/Al–O–P linkages independently of calcination history.^14^

Across the multicomponent system, a metal-specific response was observed, spanning effective removal, near-invariance, and apparent release. Fe exhibited the clearest removal signal, decreasing from 6.19 mg L^–1^ to 1.00–1.95 mg L^–1^ after contact with the lattices, depending on the calcination strategy. Interestingly, the uncalcined sample produced the lowest residual concentration, which is notable because at pH ≈ 3 classical surface adsorption of cations is often suppressed by proton competition. Therefore, the improved Fe decrease for the uncalcined lattice likely reflects not only metal retention onto phosphate-bearing sites, but also solution conditioning effects, such as the release of phosphate species that can promote Fe–phosphate association and/or precipitation within or near the porous network. We emphasize that this phosphate-mediated mechanism is presented as a hypothesis; direct quantification of phosphate release as a function of calcination was not performed and is recommended as future work to confirm it. This interpretation is consistent with a companion investigation on 3D-printed PAGP–PLU/AC composites exposed to comparable acidic conditions (pH ≈ 3), which proposed phosphate-mediated interfacial precipitation of Fe^3+^ as a strengite-like FePO_4_·2H_2_O phase as the dominant immobilization pathway, supported by thermodynamic supersaturation arguments and by the proton-releasing pH evolution observed during contact.^14^

In contrast, Cu remained essentially unchanged (3.13–3.30 mg L^–1^) and Mn slightly increased after contact. Both trends are consistent with the expected behavior at pH ≈ 3, where proton competition and sulfate complexation can strongly limit net uptake. Under such conditions, any weak retention signal can be easily masked by subtle dissolution from the matrix or by speciation shifts. A similar interpretation applies to Zn, which increased from 11.99 mg L^–1^ to 13.44–13.94 mg L^–1^, indicating net leaching rather than removal by retention from the solution. Because Zn was present in the initial solution, this increase most plausibly reflects a combination of limited adsorption at low pH and additional Zn contribution from the lattice (e.g., impurity-derived leaching or desorption of loosely retained Zn originally associated with the solid), reinforcing the observation that matrix stability is central when interpreting metal removal under highly acidic conditions.

Since leaching occurred, in Table 4 are shown the liquid metal uptake for the materials of Table 3, calculated by subtracting the value of Ci from the one for C15h. Positive values indicate the net leaching from the material to the solution and negative values indicate the net removal of the metal in solution by the geopolymer.

**Table 4:** Net Concentrations of Metal Ions Leached from the Lattice to the Solution (+) or Removed from the Solution (−) for PAGP–PLU Lattices Subjected to Different Calcination Strategies

Metal ion	PAGP–PLU_0.25_	PAGP–PLU_0.25/350 °C_	PAGP–PLU_0.25/450 °C_	PAGP–PLU_0.25/550 °C_
Al (mg L^–1^)	38.63 ± 0.42	22.73 ± 1.98	19.93 ± 0.28	9.88 ± 0.92
Ba (μg L^–1^)	63.37 ± 0.72	48.14 ± 8.52	48.47 ± 1.14	41.47 ± 3.39
Cr (μg L^–1^)	7.48 ± 0.34	2.80 ± 0.36	3.10 ± 0.61	1.75 ± 0.24
Cu (mg L^–1^)	0.07 ± 0.07	–0.10 ± 0.05	–0.02 ± 0.06	0.05 ± 0.06
Fe (mg L^–1^)	–5.19 ± 0.13	–4.26 ± 0.18	–4.38 ± 0.18	–4.24 ± 0.13
Mn (mg L^–1^)	0.33 ± 0.09	0.25 ± 0.12	0.17 ± 0.04	0.20 ± 0.04
Ni (μg L^–1^)	25.29 ± 5.94	24.71 ± 3.61	12.61 ± 1.11	10.16 ± 1.51
V (μg L^–1^)	21.98 ± 0.76	10.37 ± 1.11	13.10 ± 0.51	7.54 ± 0.31
Zn (mg L^–1^)	1.95 ± 0.56	1.70 ± 0.64	1.90 ± 0.64	1.45 ± 0.35

Taken together, calcination did not produce a single-direction improvement in metal removal performance if judged strictly by net removal of the target AMD metals (Fe, Al, Zn, Mn, Cu): Fe removal was strongest for the uncalcined lattice, and Cu, Zn and Mn were largely unaffected. However, calcination clearly enhanced chemical stability, evidenced by the systematic reduction of Al/Ni/V release with increasing temperature, an essential prerequisite for practical AMD remediation, since geopolymer leaching can compromise treated-water quality. Practically, the results suggest that calcination shifts the material behavior from a more reactive, chemically noninert lattice (greater interaction with Fe but higher release of Al and trace elements) toward a more stabilized lattice at ≥450–550 °C (reduced leaching but limited net uptake under strongly acidic conditions). This trade-off is consistent with the known sensitivity of metal removal at low pH and the susceptibility of aluminosilicate/phosphate networks to acid attack.

## 4 Conclusions

DIW-printed PAGP–PLU lattices displayed calcination-dependent trade-offs linking composition, multiscale porosity, mechanical integrity, and behavior in acidic multicomponent solutions. XRD/FTIR showed that heating from 350 to 550 °C progressively removes the PLU template and drives dehydration/condensation of the phosphate-bearing network, in agreement with the reported consolidation pathways of acid–based geopolymers. Although calcination markedly increased bulk porosity and promoted mesopore opening (maximum BJH pore diameter and pore volume at 450 °C), the BET_SSA_ increased only slightly (4.89 to 6.53–6.87 m^2^ g^–1^), indicating that the micro/mesoporous surface probed by N_2_ physisorption is comparatively resilient to thermal treatment and that the dominant porosity changes arise mainly from larger voids/structural macroporosity not fully quantified by gas sorption. This microstructure evolution imposed a clear strength–porosity relationship: 350 °C enhanced compressive strength while maintaining moderate porosity, whereas ≥450 °C produced highly porous lattices (61–63%) with a sharp loss in strength (∼5–6 MPa). In simulated AMD (pH 2.96), calcination did not consistently improve net removal of all target metals, yet it systematically decreased the release of framework/trace species (especially Al, Ni, and V), demonstrating improved chemical stability, an essential requirement for deployment in strongly acidic waters. Finally, these results indicate that calcination temperature should therefore be selected according to the desired balance between mechanical robustness and porosity. The specific advance of this work is a processing, structure, and stability relationship for DIW-printed phosphate-activated geopolymers, which shows that the calcination schedule can be selected to favor either mechanical robustness (350 °C) or higher porosity with reduced leaching (at or above 450 °C). Future work should include leaching tests in ultrapure water and the quantification of phosphate release to further isolate the dissolution behavior of the framework.
